# The KT Jeang Retrovirology prize 2021: Peter Cherepanov

**DOI:** 10.1186/s12977-021-00573-1

**Published:** 2021-09-27

**Authors:** Retrovirology Editorial

**Affiliations:** London, UK


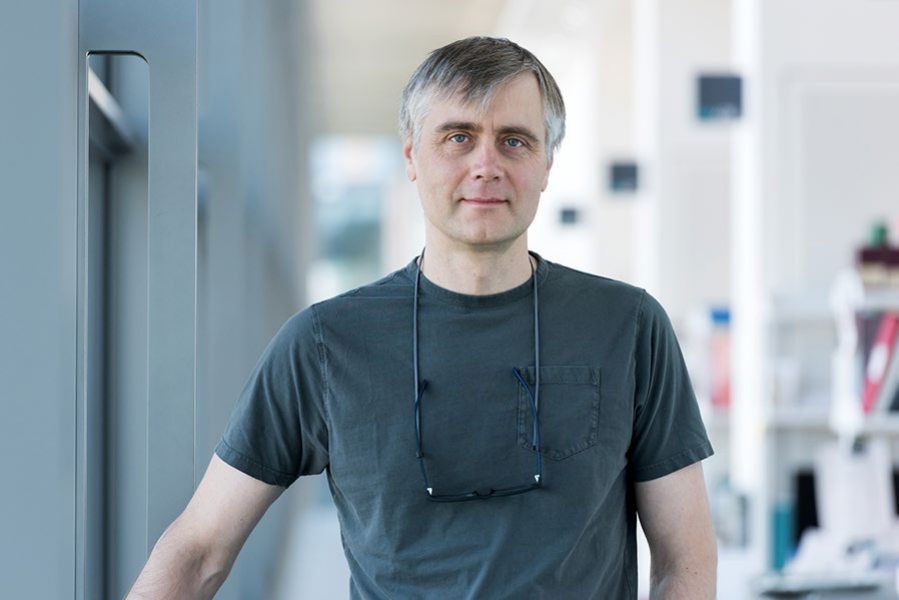
Peter Cherepanov studied Chemistry and Natural Sciences at the Novosibirsk State University (USSR), which is located in Academic Town amidst dozens of research institutes. There, he was inspired by Rudolf Salganik's lecture on DNA recombination. Science job prospects were limited in 1990's Russia and Cherepanov took part in a student exchange program, which brought him to Carl von Ossietzky University (Oldenburg, Germany). The cultural shock of moving from early post-Soviet Russia to West Germany notwithstanding, he was extremely fortunate to join the laboratory of Wilfried Wackernagel, whose research focused on DNA recombination and horizontal gene transfer in bacteria. There, Cherepanov was taught many essential bench techniques that proved indispensable for his future career. He was grateful to be given a chance to develop a small research project to adapt a budding yeast site-specific recombinase for surgery on the bacterial chromosome, which would become a widely used tool in bacterial genetics [1].

In 1995, Cherepanov moved to the Rega Institute (Katholieke Universiteit Leuven), where he joined Zeger Debyser as his first PhD student. Headed at the time by Erik De Clercq, the Belgian research institute was responsible for many remarkable contributions to the fight against AIDS, including the discovery of Tenofovir and the class of non-nucleoside HIV-1 reverse transcriptase inhibitors. The early success of reverse transcriptase and protease inhibitors greatly encouraged the global efforts to discover small molecule antagonists of the third and the last HIV-1 enzyme, integrase (IN), which inserts the ends of the viral DNA reverse transcript into a host cell chromosome [reviewed in [2]]. However, HIV-1 IN remained a poorly understood protein, which proved to be one of the least cooperative structural biology targets that would frustrate crystallographers for years to come.

During his PhD, Cherepanov was developing biochemical assays and characterising early IN inhibitor candidates. As part of the effort by the Debyser laboratory to make the drug screening assays more predictive of their activity in vivo, he established stable cell lines expressing HIV-1 IN [3, 4]. Having defended his PhD thesis in 2000, Cherepanov spent over a year painstakingly characterizing biochemical properties of HIV-1 IN in human cell extracts, which led him to identify LEDGF/p75 as the major cellular IN-binding partner [4]. In this landmark study, he showed that LEDGF/p75 interacted directly with HIV-1 IN and dramatically stimulated its enzymatic activity in vitro. Together with his soon-to-become spouse, Goedele Maertens, they showed that the host factor tethered HIV-1 IN to chromosomes in live human cells [5]. Accordingly, LEDGF/p75 would later emerge as a targeting factor determining the distribution of HIV-1 integration sites in infected cells [6, 7] [reviewed in [2, 8]].

In 2003, Cherepanov moved to the laboratory of Alan Engelman at the Dana-Farber Cancer Institute (Boston, USA). The two-year postdoc would become a transformative experience for Cherepanov. Affiliated with Harvard Medical School, this was an ideal place to collaborate with some of the best people in their fields. Here, Cherepanov and Engelman worked with Gerhard Wagner's and Tom Ellenberger's laboratories, which led to the NMR structure of the IN-binding domain of LEDGF/p75 [9] and a co-crystal structure of its complex with the catalytic core domain of HIV-1 IN [10]. This latter structure revealed the crucial details of the virus-host interface, which later would inform the design of competitive inhibitors of the IN-LEDGF/p75 interaction by Debyser and colleagues in Leuven [11].

In 2005, Cherepanov was recruited to Imperial College London (UK) as a Senior Lecturer within the Section of Infectious Diseases, headed by Myra McClure. There, after a blissful year working on the bench as a sole member of the “Cherepanov group” [12], he was fortunate to acquire funding from Tibotec Pharmaceuticals (now part of Janssen Pharmaceuticals, Johnson & Johnson) and the UK Medical Research Council. Now properly staffed, the Cherepanov lab worked on characterising the complete lentiviral IN-LEDGF/p75 interface and carefully evaluating a range of retroviral IN proteins for structural characterization [12–14]. His long-term view was to find a more “agreeable” retroviral IN protein, which could be persuaded to yield structural insights into the assembly of the functional IN-DNA complexes, or the “intasomes”. The first real breakthrough came when the group discovered that IN from the prototype foamy virus (PFV, a spumavirus), was highly soluble and active in vitro [15]. The next two years were spent on attempts to assemble and crystallise the PFV intasome. Although crystals were identified regularly, they invariably failed to diffract X-rays. By the time a useful crystal form was discovered, Saumya Gupta, a junior technician brave enough to commit to this project, tested over 40,000 individual crystallization conditions. After an arduous and seemingly endless crystal screening campaign, all data arrived suddenly during a very brief session at the Diamond Light Source synchrotron. The result was the first structure of the retroviral intasome, built and refined by Steve Hare [16]. Very different from any model proposed previously, the crystal structure revealed how a tetramer of PFV IN synapses a pair of viral DNA ends and how a functional IN active site forms. Moreover, because the active sites of PFV and HIV-1 INs are sufficiently similar, the crystals allowed the group to study the mode of action of the class of IN inhibitors that are known as IN strand transfer inhibitors (INSTIs) [16–19]. The resulting structures revealed that these compounds displace the 3'-terminal viral DNA nucleotide from the IN active site, effectively disarming the intasome.

Using X-ray crystallography, Goedele Maertens in the lab visualised how the intasome binds and deforms target DNA to carry out the insertion of viral 3' DNA ends across an expanded major groove, and how it avoids reversal of the strand transfer step [20]. Retroviral IN enzymatic activity strictly depends on the presence of divalent metal cations (Mg^2+^ or Mn^2+^). Taking advantage of this property, Goedele Maertens and Steve Hare were able to freeze-trap the Michaelis complexes of the intasome primed for catalysis upon brief soaks of the crystals in the presence of the essential metal ion co-factors. The results revealed hitherto unappreciated DNA substrate mimicry by the INSTIs, informing ways to improve this important class of clinical compounds [21].

In 2011, the Cherepanov laboratory moved to Clare Hall Laboratories of the London Research Institute (Cancer Research UK), which in 2015 became one of the founding institutions of the Francis Crick Institute. The target of retroviral integration is cellular chromosomal DNA, which exists in the form of nucleosomal arrays. It had been well established that retroviruses do integrate into nucleosomes, but it was not understood how the integration machinery could access DNA wrapped around a histone octamer. Daniel Maskell, a postdoc in the lab, was able to identify a human nucleosome that could form a stable asymmetric complex with intasome that survived purification. In collaboration with the Alessandro Costa lab, the groups were able to determine a cryo-EM structure of the PFV intasome bound to a human nucleosome. The structure revealed that the intasome makes extensive interactions with the nucleosome, which allow it to peel the DNA from the histone octamer core to force it into optimal conformation for strand transfer [22]. The structure was validated using very clever compatible pairs of mutations designed by Steve Hare and virology experiments in the Engelman lab.

Although the PFV intasome was very informative of the fundamentals of retroviral integration, PFV IN outside of the immediate active site is highly divergent from its HIV-1 counterpart. To close this knowledge gap, the Cherepanov lab characterized several lentiviral INs that are considerably closer to the HIV-1 protein. Eventually, Daniel Maskell and Allison Ballandras-Colas in the group were able to generate high-quality preparations of the intasome from maedi-visna virus, an ovine lentivirus. In collaboration with Costa's lab, they refined a cryo-EM structure of this intasome, which revealed an assembly containing sixteen IN subunits, arranged as a tetramer-of-tetramers [23]. Embedded within it, was a core assembly structurally equivalent to the PFV intasome. Harbouring four IN chains, the conserved intasome core (CIC) has been observed in all other retroviral intasomes characterized to-date [2]. The expansion of the lentiviral intasome structure may allow it to form multivalent interactions with target chromatin, potentially making it more sensitive to the local epigenetic environment.

The low level of amino acid sequence identity between PFV and HIV-1 INs greatly complicates the studies of drug resistance associated with mutations in and around the HIV-1 IN active site. To circumvent this problem, Nicola Cook, a scientific officer in the Cherepanov group, evaluated approximately 20 simian immunodeficiency virus IN proteins. She discovered that the IN from the red-capped mangabey virus (SIV_rcm_), which is highly similar to HIV-1 IN, readily assembled into intasomes that could be embedded in amorphous ice. Wen Li and Alan Engelman established the essential SIV_rcm_ infectivity assays to validate the virus as a model for HIV-1 drug resistance studies, and Cherepanov refined the SIV_rcm_ intasome structure at a near atomic resolution [24]. The structural data and molecular dynamics computations by Magd Badaoui and Denes Berta in Edina Rosta’s lab collectively identified features of the second-generation INSTIs responsible for their improved antiviral activity and showed that commonly occurring INSTI-resistance mutations act by destabilising the metal chelating cluster within the IN active site [24].

In addition to integration, Cherepanov has a broad interest in virus-host interactions, where his lab made seminal contributions to the structural basis of nuclear import of CPSF6 and other SR proteins by Transportin 3 (Goedele Maertens and Nicola Cook, in collaboration with the Fassati and Engelman labs) [25], the mechanism of tethering PFV capsids to chromosomes for integration (Paul Lesbats, in collaboration with the Lindemann and Engelman labs) [26], determination of the first structures of the SERINC family of HIV-1 restriction factors (Valerie Pye, in collaboration with the Pizzato lab) [27], and the unexpected discovery of modulation of SARS-CoV-2 spike neutralisation by heme metabolites (Annachiara Rosa and Valerie Pye in collaboration with the Kassiotis, Doores and McCoy labs) [28].
